# The Importance of Improving the Quality of Care Among HIV/AIDS Hospitalizations in Portugal

**DOI:** 10.3389/fpubh.2019.00266

**Published:** 2019-09-13

**Authors:** Ahmed N. Shaaban, Maria Rosario O. Martins

**Affiliations:** ^1^Global Health and Tropical Medicine, Institute of Hygiene and Tropical Medicine, NOVA University of Lisbon, Lisbon, Portugal; ^2^EPIUnit—Instituto de Saúde Pública, Universidade Do Porto, Porto, Portugal

**Keywords:** HIV/AIDS, quality of care (measurement), length of stay (LOS), 30-day readmission, hospital performance indicators

HIV affects almost 37.9 million persons globally, and an estimated 1.7 million new HIV infections occur in 2018 (1). While HIV/AIDS is taking a devastating toll on populations' health, lives and families, the disease is imposing a serious economic burden on governments (2–4) being classified as the greatest single financial burden on healthcare systems globally (5). This burden is predominantly due to the high payments of antiretroviral therapy (ART), hospitalizations, and associated opportunistic infections treatment (6, 7). In Portugal, HIV continues to be a major public health concern and HIV prevalence is among the highest in Europe (8, 9) with 41,000 individuals who are living with HIV, representing 0.5% of the total population (10) (see Table 1 for an informative overview of HIV/AIDS in Portugal). The country also still records annual rates of new HIV/AIDS diagnosis, which have been classified among the highest in the European Union (EU) (8). Admissions among HIV/AIDS patients still pose considerable challenges to the Portuguese national health system (5, 11). In Portugal, hospitalizations related to HIV/AIDS are some of the most expensive with an average daily cost of €825 and an average length of stay of 23 days, placing HIV/AIDS as the second greatest Major Diagnostic Category (MDC) (5, 12). In addition, and after the financial crisis that hit Portugal in 2011, the country went through strict fiscal austerity that resulted in budget cuts, reduction of spending on sensitive health sectors, and restructuring numerous public entities including the National AIDS Program (NAP) (12–14). It is important to know that the average cost of HIV treatment in Portugal is about 14,000 €/patient per year (6). The main cost driver is the antiretroviral medications (€ 9,598), followed by hospitalizations (€ 1,323). Treatment costs grow with the severity of disease from € 11,901, with a CD4 count more than 500, to € 23,351, with a CD4 count <50 (6). In other words, while cost related to antiretroviral remains constant over the course of the disease, the cost progression remains mainly linked to the associated hospitalizations and admissions related to HIV. Moreover, the shift of HIV infection from a fatal disease into a chronic illness carries substantial challenges to the health system. The introduction of antiretroviral therapy (ART) has dramatically increased the life expectancy of HIV patients (15–17). This modification in the natural evolution of HIV infection has led to a substantial increase in the financial burden and cost due to the net increase in the number of people living with HIV and the associated life-long treatment and comorbidities (16, 18).

**Table 1:** HIV/AIDS estimates in Portugal.

Adults aged 15 and over living with HIV	41,000 (CI: 36,000–46,000)
Adult aged 15–49 HIV prevalence rate	0.5 (CI: 0.4–0.5)
HIV incidence per 1,000 population (adults 15–49)	0.10
People living with HIV who are on ART	37,000
Percent of people living with HIV who are on ART	90 (CI: 78–95)
Average cost of HIV treatment per year	14,277 €/patient^*^

Sources: UNAIDS,

* Perelman et al. (6).

*CI, Confidence Interval*.

In this context, one possible solution to overcome this associated economic burden is to increase value in healthcare by integrating quality measures of hospitals' performance while reducing the costs of healthcare. Previous reviews supported the evidence that there is great potential for decreasing costs by targeting deficiencies in quality, and accordingly we can maximize the benefits given the available resources (19–24). Two important quality measures had obtained growing attention as a benchmark indicator for measuring hospital's performance, thirty-day readmission rate, and length of stay (LOS) (22, 25–28). However, despite being optimal methods for assessing hospital's performance, there remains a scarcity of research pertaining to the factors that can influence these quality indicators, especially when it comes to assessing hospitalizations among HIV/AIDS patients in Portugal. Thirty-day hospital readmission is defined as an episode in which a patient is readmitted within 30 days from the last discharge. Early readmission rates have increasingly been used as an outcome measure in health services research and as a quality benchmark for health systems (29–31). However, although often preventable, early readmissions have been recognized as frequent and costly events (32–34). For example, in the United States, one in five Medicare beneficiaries has 30-days readmission, with a cost of around $26 billion per year (34, 35). Accordingly, hospital readmission rates were incorporated in the reimbursement decisions for several programs, in which the health systems penalize hospitals with higher than expected readmission rates (33, 36).

The second quality indicator is length of stay which is defined as the number of days a patient is hospitalized in relation to the admission diagnosis and it had been widely used to evaluate the effect of implementing patient group related reimbursement systems in the form of Diagnosis Related Groups systems (DRG) is length of stay (37, 38). This quality indicator has been recommended as an important outcome measure for quality improvement activities (28). Using length of stay as a hospital performance measure will allow us to impact cost and quality through payment incentives for hospitals or health care providers. For example, if a hospital reduces length of stay and accordingly the other associated resources and costs, the hospitals will be more efficient through maintaining a higher marginal return on each per admission payment (22).

The Portuguese national database of admissions among HIV patients can be obtained for research purposes from the Administration of the Health System (ACSS) (39). These data are anonymous, refers to the Diagnosis Related Groups (DRGs), and each record corresponds to a discharge episode and contains information collected while the patients were admitted to the hospital. These data include information about length of stay as well as information collected during the hospitalization that include socio-demographic characteristics (age, sex, region of residence), dates of hospitalization and discharge, Index hospitalization (admission type (urgent or scheduled), type of intervention (surgical or medical), type of diagnosis (primary and secondary diagnoses), type of procedures during the hospitalization), prior health care utilization (mode of transfer, destination after discharge), outcome at discharge (alive or deceased), coverage by the national health system (Yes/No). To determine 30-day readmission for each hospitalization, a unique fictional code included in the data can be used since it allows determining how many episodes correspond to the same user, in the same institution. This fictional code does not identify the user nor allow its identification afterward. Accordingly, readmission episodes and the time span between the readmission and the last discharge can be calculated for each hospitalization. accordingly, The variable of interest can be created as follows: Y = 0 if hospitalizations without subsequent 30-day readmission, Y = 1 if hospitalizations with subsequent 30-day readmission(s). Univariate and multivariate logistic models can be estimated afterward to identify the determinants of hospitalizations with subsequent 30-day readmission.

Regarding length of stay, each hospitalization is associated with a record that refers to the number of days each person remains at hospital as a count data. However, giving the statistical nature of length of stay as count data, caution should be taken when handling such data with count distribution [for an informative overview of count distribution see (40)]. Using the most common techniques, namely the ordinary least squares (OLS) or logistic regression to handle a dependent variable with positive skewness as occurs in LOS, will violate the fundamental assumptions behind each technique (41, 42). Accordingly, this may result in biased and inefficient estimates and produce results that do not accurately reflect the observed data (41, 42). Fortunately, count statistical techniques (Poisson, negative binomial, Zero Inflated Poisson, and Zero Inflated Negative Binomial models) have been developed to handle count data on a dependent variable and can replace these suboptimal statistical strategies (43, 44). Using these count regression techniques will allow us to accurately determine the factors that can push length of stay further. Moreover, the conceptual and statistical advantages of each count model should be illustrated precisely since the accuracy and nature of results tend to vary depending on the specific model utilized.

This opinion contributes to the attempts on reducing the economic burden of HIV in Portugal, which is in line with Portugal's policy of cost reduction as a target to stabilize the economic situation. Our opinion point to other concerns that need to be considered: integration of quality measures as a method of evaluating hospitals' performance is crucial in the light of limited resources and should be considered as a national priority. A considerable work should be devoted to controlling and investigating the factors which tend to push 30-day admission rate and length of stay expenses further. The statistical nature of quality measures requires a deep understanding of the appropriate statistical models that should be used to avoid biased estimates. Finally, there is a potential in policy decision-making concerning the optimal use of limited resources and as a first step, we should deeply investigate the determinants of 30-days readmission and length of stay among HIV patients in Portugal.

## Author Contributions

AS conceived the work, reviewed literature, and wrote the manuscript. MM supervised the work and wrote the manuscript. All the authors have agreed on the final version of the manuscript.

### Conflict of Interest Statement

The authors declare that the research was conducted in the absence of any commercial or financial relationships that could be construed as a potential conflict of interest.
